# Evaluation of a complex healthcare intervention to increase smoking cessation in pregnant women: interrupted time series analysis with economic evaluation

**DOI:** 10.1136/tobaccocontrol-2016-053476

**Published:** 2017-02-15

**Authors:** Ruth Bell, Svetlana V Glinianaia, Zelda van der Waal, Andrew Close, Eoin Moloney, Susan Jones, Vera Araújo-Soares, Sharon Hamilton, Eugene MG Milne, Janet Shucksmith, Luke Vale, Martyn Willmore, Martin White, Steven Rushton

**Affiliations:** 1 Institute of Health and Society, Newcastle University, Newcastle upon Tyne, UK; 2 School of Biology, Newcastle University, Newcastle upon Tyne, UK; 3 Health and Social Care Institute, Teesside University, Middlesbrough, UK; 4 Newcastle City Council, Newcastle upon Tyne, UK; 5 Fresh, Smoke Free North East, Durham, UK; 6 MRC Epidemiology Unit, School of Clinical Medicine, University of Cambridge, Cambridge, UK

**Keywords:** smoking cessation, pregnancy, guidelines, implementation, natural experimental evaluation, economic evaluation

## Abstract

**Objectives:**

To evaluate the effectiveness of a complex intervention to improve referral and treatment of pregnant smokers in routine practice, and to assess the incremental costs to the National Health Service (NHS) per additional woman quitting smoking.

**Design:**

Interrupted time series analysis of routine data before and after introducing the intervention, within-study economic evaluation.

**Setting:**

Eight acute NHS hospital trusts and 12 local authority areas in North East England.

**Participants:**

37 726 records of singleton delivery including 10 594 to mothers classified as smoking during pregnancy.

**Interventions:**

A package of measures implemented in trusts and smoking cessation services, aimed at increasing the proportion of pregnant smokers quitting during pregnancy, comprising skills training for healthcare and smoking cessation staff; universal carbon monoxide monitoring with routine opt-out referral for smoking cessation support; provision of carbon monoxide monitors and supporting materials; and an explicit referral pathway and follow-up protocol.

**Main outcome measures:**

Referrals to smoking cessation services; probability of quitting smoking during pregnancy; additional costs to health services; incremental cost per additional woman quitting.

**Results:**

After introduction of the intervention, the referral rate increased more than twofold (incidence rate ratio=2.47, 95% CI 2.16 to 2.81) and the probability of quitting by delivery increased (adjusted OR=1.81, 95% CI 1.54 to 2.12). The additional cost per delivery was £31 and the incremental cost per additional quit was £952; 31 pregnant women needed to be treated for each additional quitter.

**Conclusions:**

The implementation of a system-wide complex healthcare intervention was associated with significant increase in rates of quitting by delivery.

## Introduction

Maternal tobacco smoking during pregnancy is a global public health problem. In high-income countries smoking is a major cause of stillbirth^1 2^ and low birth weight.^3^ Interventions to promote smoking cessation during pregnancy are effective and cost-effective in improving pregnancy and other health outcomes.^4–7^ In the UK, national guidance recommends routine carbon monoxide (CO) monitoring at antenatal visits to identify smokers, and opt-out referral to smoking cessation support (The National Institute for Health and Care Excellence).^8^ However, wide variations in rates of smoking at the time of delivery persist.^9^

The smoking at delivery rate in North East England is persistently higher than the national average, and prior to this study in 2012 was around 20%.^9^ We identified that implementation of national guidance varied between local maternity units. Availability of CO monitors, midwives’ doubts about the effectiveness of offering smoking cessation interventions and concerns about damaging relationships with pregnant smokers were barriers to implementation.^10^ To address these issues, a complex intervention focused on improving implementation of national guidance was commissioned for the region via the tobacco control office for North East England (Fresh, www.freshne.com). The intervention focused on systematic implementation of routine biochemical validation and opt-out referral of pregnant smokers to smoking cessation services. Two recent studies of opt-out referral have reported contrasting findings. One found an increase in referrals to smoking cessation services but no impact on quit rates.^11^ The second, in a single hospital trust using a similar intervention to this study, found a doubling of referrals and of 4-week quit rates.^12^

We aimed to evaluate the effect of the introduction of this intervention across a regional health system on quit rates, and to estimate the associated costs.

## Methods

### Design and study population

We conducted an interrupted time series analysis of longitudinal data collected before and after introducing the intervention across North East England, within eight National Health Service (NHS) hospital trusts providing maternity services and 12 local authorities commissioning smoking cessation services. We analysed the pathway from identification of smoking at first antenatal visit, through referral to smoking cessation services, quitting during pregnancy and subsequent birth outcomes before and after the introduction of the intervention.

### Intervention

**Figure 1:**
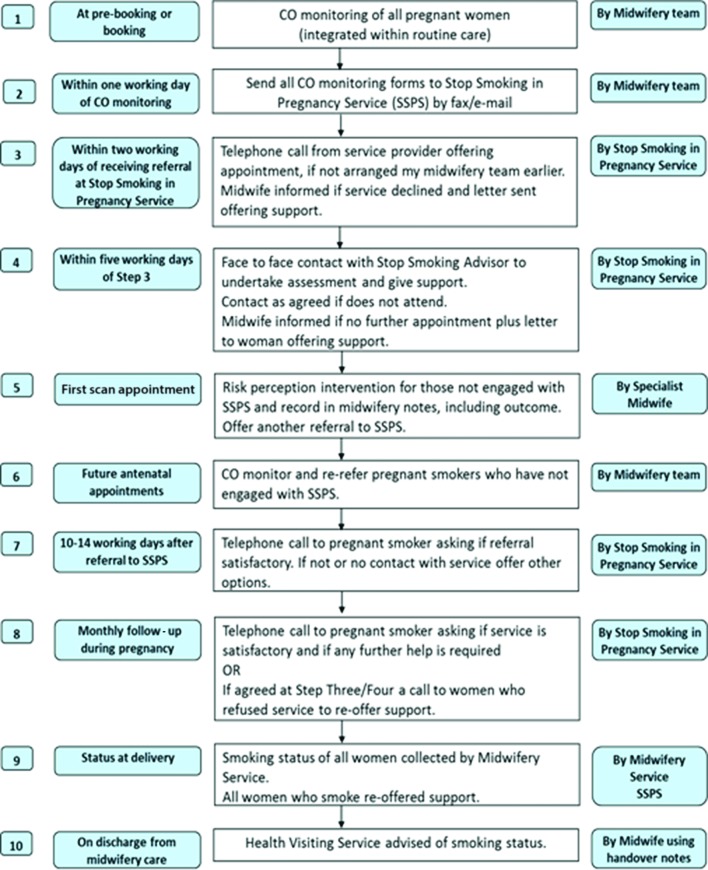
Intervention referral pathway.

BabyClear is a complex intervention,^13^ developed by the Tobacco Control Collaborating Centre, part of Improving Performance in Practice. It comprises a package of measures designed to support the implementation of national guidance (figure 1; appendix 1). In England, responsibility for commissioning smoking cessation services lies with local authorities, while responsibility for commissioning maternity services lies with NHS England. Antenatal care is delivered by NHS trusts but smoking cessation services may be delivered by a range of community-based providers. The intervention was designed to strengthen links between these two services. Skills training was provided for maternity staff, smoking cessation advisors and administrators within smoking cessation services. Midwives were trained to implement universal CO monitoring at first antenatal appointment, with routine opt-out referral for smoking cessation advice for any woman with a CO recording above four parts per million (ppm).^14^ Communication skills, including approaches to introduce CO monitoring to women, were developed during training. CO monitors and referral forms were provided to all participating trusts, and an explicit referral pathway and follow-up protocol for smoking cessation services were introduced. The intervention was not developed with an explicit theoretical underpinning, but incorporated recognised behaviour change techniques^15^ and was felt to address the needs identified by our previous work.^10^ The core intervention, comprising skills training, supporting materials and referral pathway, was introduced between November 2012 and July 2013. The full babyClear package included an additional enhanced ‘risk perception’ intervention delivered at first trimester ultrasound scan appointment, but implementation of this component was delayed and we do not evaluate its impact here. A parallel process evaluation was conducted and is reported elsewhere.

10.1136/tobaccocontrol-2016-053476.supp1 Supplementary file

### Data sources

We obtained electronic records of deliveries from trusts, encompassing a pre-intervention and post-intervention period with at least 4 months of data post-intervention for each trust. Data on referrals and quit attempts were obtained from smoking cessation services and linked to delivery data using maternal NHS number where available, or by mothers’ date of birth and postcode. Once matched, records were anonymised and combined into a single database, and multiple pregnancies were excluded. Clinical and demographic variables obtained from maternity records included birth weight, gestation at delivery, infant sex, outcome of pregnancy, smoking status of mother at booking and delivery, maternal height, weight and body mass index, maternal ethnic group, maternal age and Index of Multiple Deprivation (IMD, an area-based measure assigned using maternal postcode). 16 Data requested from smoking cessation services included referral dates, appointments, quit dates and quit status at 4 weeks. Our data therefore described demographic details of mothers, smoking status during pregnancy and at delivery, engagement with smoking cessation services and outcomes before and after the introduction of the intervention.

### Patient involvement

We used routine data sources for this evaluation. Patients were not involved in the design or conduct of the study.

### Variable definitions

We classified maternal smoking status during pregnancy on the basis of variables from maternity units or smoking cessation services which indicated smoking at any time during pregnancy (appendix 2). Smoking status at delivery was defined using routinely collected data from maternity units, definition and collection of which is mandated by the Department of Health.^9^ Quitting during pregnancy was defined as any mother classified as smoking during pregnancy but recorded as a non-smoker at delivery. All other women were classified as non-smokers throughout pregnancy.

10.1136/tobaccocontrol-2016-053476.supp2 Supplementary file

The start of the intervention was defined as the month during which the initial midwives’ training session was delivered in each trust. We classified deliveries as ‘before intervention’ or ‘after intervention’ depending on when the pregnancy reached 11 weeks’ calculated gestation.

Referral for smoking cessation advice was defined as a delivery with any of the following recorded: date of referral; being sent information or contacted by a smoking cessation service; an appointment booked or attended with the smoking cessation service; a record of a quit date being set; or any record of smoking status recorded for ‘quit at 4 weeks’. We created monthly counts of referrals to assess referral rates over time.

We collated information on how the intervention was implemented in each trust as part of a parallel process evaluation and created variables to describe the intervention. The variables were dates (month) of additional training sessions for midwives who had missed the initial training and enhanced arrangements for initial contact with smokers.

The range of IMD scores within the cohort were categorised into five groups based on internal quintiles. The first quintile represented the least deprived 20% of scores within the cohort (IMD 0–16). The central quintiles (range of scores 17–64) were grouped together; the final quintile included the most deprived 20% of scores (65–80). The groups were restructured as ordinal variables 1–3 within the final model.

### Statistical analysis

We created a complete case dataset for use in the analyses. We modelled two outcomes that we hypothesised would be influenced by the intervention: (1) change in the monthly referrals to smoking cessation service per trust and (2) change in individual maternal quit rates; within the whole cohort, we investigated the impact of quitting during pregnancy on birth weight (appendix 2).

We used a mixed-effects modelling approach to investigate how the intervention impacted on the monthly referral rate (model 1). We hypothesised that there would be variation between trusts, specifically in relation to the initial level of recorded referral rates, and so modelled trust as a random intercept and compared it with a simpler linear model without random effects. A categorical variable was used to compare referral rates pre-intervention and post-intervention. We included time since the introduction of the intervention in each trust (continuous variable) as a proxy for changes that might have arisen through changes in efficiency as implementation progressed. We also estimated the effect of the initial 4 months since the intervention (categorical variable). To control for potential variation in baseline referral rates through time for each trust, we included random effects within the model that incorporated a random intercept and slope. This allowed the model to estimate the deviation in overall referral rate for each trust (captured by the random intercept) while also accepting that referral rates achieved by individual trusts also varied through time (random slope).

The effect of variation between trusts with regard to service provision was also examined. Variables describing the initial contact with smokers, and additional training in any given month, were included. We used a stepwise reduction approach to identify the most parsimonious model at each stage and analysed the error.

We investigated the effects of the intervention on the probability that an individual mother would quit smoking before delivery in a similar way, using a logistic regression mixed-effects modelling approach with random intercept for trust (model 2). We used quitting as a binary response variable (yes/no) for individual pregnancies. We adjusted for maternal ethnicity, age, parity and deprivation.

We investigated the effects of quitting on birth weight in the whole cohort, using linear mixed-effects models with trust as random effect (model 3), with the same set of maternal demographic covariates used in model 2. We did not use intervention status as a covariate in this model. We investigated the effects of quitting and smoking on birth weight as a continuous outcome.

All analyses were undertaken in R^17^ using the Linear and Nonlinear Mixed Effects Models statistical package.^18^

### Economic analysis

We estimated the additional cost to the NHS over a 5-year time horizon. Costs included those for training of staff, investment in equipment and consumables and changes in workload, and were costed using routine sources. Costs are presented as an average for the participating trusts and are reported in UK pounds sterling (£) for 2013. Data on the mean number of pregnancies and the number of additional quitters per trust were calculated by combining data on the average quit rate per trust before implementation, combined with the estimated adjusted OR (aOR) for quitting after implementation and data on smoking prevalence in the entire cohort. These data were combined with costs to produce the incremental cost, and numbers needed to treat, per additional quit (appendix 3).

10.1136/tobaccocontrol-2016-053476.supp3 Supplementary file

## Results

### Study population

There were 37 726 records of singleton delivery across the eight trusts. Twenty eight per cent of mothers were classified as smokers. Table 1 shows the characteristics of smokers and non-smokers within the cohort.

**Table 1:** Maternal characteristics of study cohort in North East England

Variable	Categories	Total in cohort (%)	Smoking status:		
			Non-Smokers	Smokers	Missing
Maternal age (years)	15–20	3501 (9.3)	1858 (6.9)	1626 (15.3)	17 (20.7)
	21–30	20 401 (54.1)	14 135 (52.3)	6226 (58.8)	40 (48.8)
	31–40	13 163 (34.9)	10 538 (39.0)	2600 (24.5)	25 (30.5)
	41+	651 (1.7)	510 (1.9)	141 (1.3)	0 (0.0)
	Missing	10 (0.0)	9 (0.0)	1 (0.0)	0 (0.0)
Parity	First child	13 476 (35.7)	10 449 (38.6)	3000 (28.3)	27 (32.9)
	Second child	11 166 (29.6)	8205 (30.3)	2942 (27.8)	19 (23.2)
	Third + child	7362 (19.5)	4540 (16.8)	2796 (26.4)	26 (31.7)
	Missing	5722 (15.2)	3856 (14.3)	1856 (17.5)	10 (12.2)
BMI (kg/m^2^)	Underweight (<20)	747 (2.0)	440 (1.6)	304 (2.9)	3 (3.7)
	Healthy (20–24.9)	12 026 (31.9)	8603 (31.8)	3389 (32.0)	34 (41.5)
	Overweight (25–29.9)	6985 (18.5)	4976 (18.4)	1990 (18.8)	19 (23.2)
	Obese (30+)	5725 (15.2)	3964 (14.7)	1752 (16.5)	9 (11.0)
	Missing	12 243 (32.5)	9067 (33.5)	3159 (29.8)	17 (20.7)
Ethnic group	White	33 614 (89.1)	23 491 (86.8)	10 041 (94.8)	82 (100.0)
	Caucasian	2736 (7.3)	2512 (9.3)	224 (2.1)	0
	Missing	1376 (3.6)	1047 (3.9)	329 (3.1)	0
SEP (categories defined by fifths of IMD score)	Least deprived (0–16)	8380 (22.2)	7142 (26.4)	1231 (11.6)	7 (8.5)
	Middle three-fifths (17–64)	27 012 (71.6)	18 408 (68.1)	8536 (80.6)	68 (82.9)
	Most deprived (65–80)	1674 (4.4)	971 (3.6)	698 (6.6)	5 (6.1)
	Missing	660 (1.7)	529 (2.0)	129 (1.2)	2 (2.4)
Total		37 726	27 050	10 594	82

BMI, body mass index; SEP, socioeconomic position (categories defined by fifths of Index of Multiple Deprivation (IMD) score).

### Model 1: effect of the intervention on referrals

**Figure 2:**
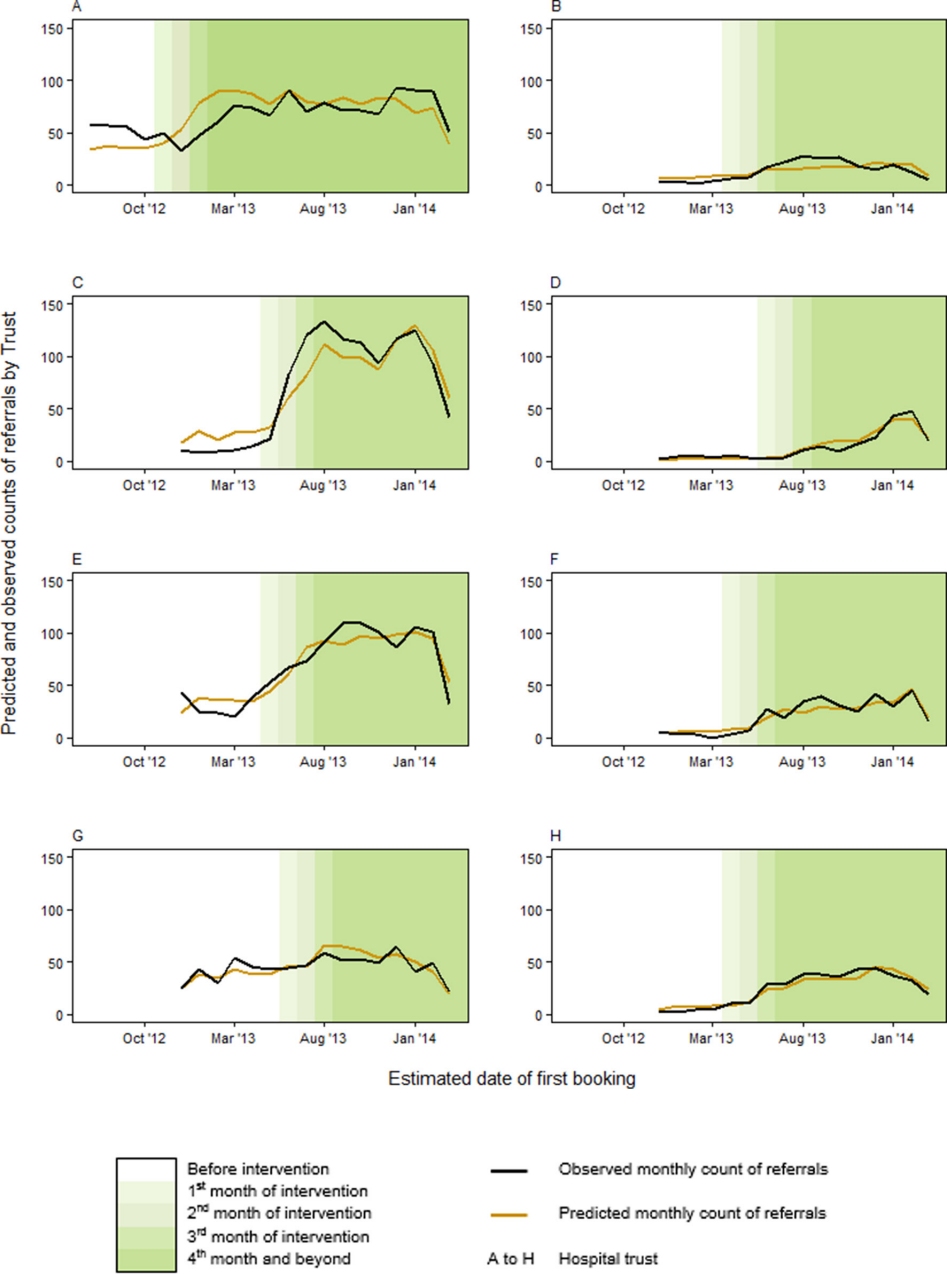
Predicted and observed monthly referral counts by trust before, during and after intervention implementation.

The referral rate increased progressively in all trusts in the first 3 months after the intervention was introduced (figure 2). The intervention was associated with a significant increase in referrals (incidence rate ratio, IRR=2.47, 95% CI: 2.16 to 2.81), beyond the fourth month following introduction (table 2). Inclusion of a random intercept for trust led to an improved model fit, indicating that there were differences in the baseline number of referrals across trusts (appendix 2). Trust effects on referral rates varied independently of intervention implementation (from IRR=1.29 in trust D to IRR=6.21 in trust G; appendix 2). Additional training sessions were associated with an increase in referrals in the month of training (IRR=1.15, 95% CI 1.06 to 1.25), as was availability of a system for enhanced initial contact with smokers (IRR=6.2, 95% CI 3.18 to 12.10 for early contact with smokers and IRR=1.78, 95% CI 1.12 to 2.84 for trusts where midwives made appointment with smoking cessation services). The fitted values of the final model reflect the observed patterns in referrals rates.

**Table 2:** Effects of the intervention (after vs before) on monthly referral counts

Variable	OR	95% CI	Pr(>|z|)
Intercept: baseline	0.02	0.01 to 0.06	<0.001
Months after implementation of intervention, compared with before			
First month	1.15	0.98 to 1.35	0.093
Second month	1.50	1.29 to 1.74	<0.001
Third month	2.14	1.87 to 2.45	<0.001
Fourth month and beyond	2.47	2.16 to 2.81	<0.001
Time since start of intervention, compared with before			
Each additional month	1.06	0.99 to 1.13	0.103
Strategy for initial contact with smokers, compared with none			
Appointment	1.78	1.116 to 2.84	<0.050
Early contact	6.21	3.183 to 12.11	<0.001
Month and availability of additional training, compared with month without training			
Month of additional training	1.15	1.064 to 1.25	<0.001

36907 valid cases included in model (2.2% cases with missing data excluded).

### Model 2: effect of the intervention on quitting during pregnancy

Introduction of the intervention was associated with a significant increase in quitting by delivery (aOR=1.81, 95% CI 1.55 to 2.12) (table 3). The odds of quitting were higher (aOR=3.23, 95% CI 2.99 to 3.71) for deliveries with a recorded referral to smoking cessation services, and if there was a record of a quit date (aOR=4.18, 95% CI 3.53 to 4.94). The odds of quitting were significantly higher following additional training, (aOR=1.02, 95% CI 1.002 to 1.03). Mothers resident in the most deprived areas were less likely to quit (aOR=0.52, 95% CI 0.42 to 0.65), as were younger mothers and those of white ethnicity.

**Table 3:** Effect of intervention (after vs before) on probability of quitting by delivery

Variable	OR	95% CI	Pr(>|z|)
Intercept: baseline	0.13	0.09 to 0.19	<0.001
After implementation of intervention, compared with before			
After intervention	1.81	1.55 to 2.12	<0.001
Ethnic group, compared with white			
Caucasian	2.52	1.837 to 3.44	<0.001
SEP category (based on IMD score) compared with middle three-fifths of distribution			
Least deprived fifth	2.75	2.386 to 3.18	<0.001
Most deprived fifth	0.4	0.42 to 0.65	<0.001
Maternal age category, compared with 21–30 years			
15–20 years	0.75	0.655 to 0.87	<0.001
31–40 years	1.43	1.29 to 1.60	<0.001
41–55 years	1.14	0.76 to 1.71	0.526
Engagement with smoking cessation services, compared with not referred			
Referred, quit date	4.18	3.53 to 4.94	<0.001
Referred, no quit date	3.33	2.99 to 3.71	<0.001
Time since start of intervention, compared with before			
Each additional month	1.02	1.00 to 1.03	<0.050

9967 valid cases included in model (6.6% cases with missing data excluded).

SEP, socioeconomic position (categories defined by fifths of Index of Multiple Deprivation (IMD) score)

### Model 3: effect of quitting on birth weight

Babies born to women who did not smoke during pregnancy were significantly heavier than those born to women who smoked throughout pregnancy (+8.04%; 95% CI+7.54% to +8.54%), equivalent to an additional 260 g for a baby born at 40 weeks gestation (term) in reference categories (table 4). Babies born to women who quit smoking by delivery had a significantly higher birth weight (equivalent to an additional 210 g at 40 weeks) compared with those whose mothers continued smoking (+6.53%, 95% CI+5.83% to+7.24%). Quitters’ babies had birth weight similar to those of non-smokers (−1.39%, 95% CI −1.94% to −0.08%).

**Table 4:** Estimates of effects of quitting smoking on log (birth weight)*

Variable	Mean (g)	95% CI	Pr(>|t|)
Intercept: baseline (40 weeks)	3233.1	3206.7 to 3259.8	<0.001
Gestational age at birth, centred at 40 weeks			
Each additional week past 40	156.8	151.0 to 162.7	<0.001
Squared	−12.6	−13.2 to −12.4	<0.001
Smoking status at delivery compared with smoker throughout			
Non-smoker	259.6	241.8 to 277.7	<0.001
Quitter	210.2	186.3 to 235.0	<0.001
Parity compared with second child			
First child	−104.4	−115.4 to −92.9	<0.001
Third or more	22.3	7.7 to 37.2	<0.005
Ethnicity compared with white			
Caucasian	−141.6	−159.4 to −123.5	<0.001
SEP (based on IMD score) compared with middle three-fifths			
Least deprived fifth	27.8	14.4 to 41.1	<0.001
Most deprived fifth	−15.5	−40.4 to 10.1	0.232
BMI compared with recommended			
Underweight (<20)	−116.7	−146.5 to −86.4	<0.001
Overweight (25–29.9)	79.5	65.7 to 93.2	<0.001
Obese (30+)	134.5	119.3 to 150.3	<0.001
Sex of baby compared with female			
Male	129.0	117.0 to 141.5	<0.001

22 826 valid cases included in model (39.5% cases with missing data excluded).

*Estimates are backtransformed to represent the actual change in birth weight of babies with 95% CI and statistical significance.

BMI, boy mass index; SEP, socioeconomic position (categories defined by fifths of Index of Multiple Deprivation (IMD) score).

### Economic evaluation

The estimated average cost of implementing the core intervention for a trust in the North East England over a 5-year period was £572 009 (appendix 3). The total number of deliveries over 5 years is estimated at 18 640 per trust, giving a cost per delivery of £30.69. The quit rate during pregnancy before the intervention was 0.0398 per delivery. Using the aOR for the effect of the intervention (aOR=1.81) gives a quit rate after intervention of 0.072 per delivery and an absolute difference in quit rate of 0.032. Thus, the incremental cost per additional quitter was £952 and the number needed to treat for each additional quitter was 31 pregnant women.

## Discussion

### Principal findings

The introduction of a system-wide intervention to promote smoking cessation during pregnancy increased referrals to smoking cessation by 2.5 times and the proportion of women quitting by delivery by nearly twofold. Quitting smoking during pregnancy was associated with a clinically important increase in birth weight.

### Strengths and limitations

Our study included more than 35 000 deliveries across a region which included eight acute hospital trusts and smoking cessation services commissioned by 12 local authorities. We evaluated the implementation of the intervention across these different organisations and localities, and showed a substantial increase in quit rates during pregnancy which is likely to have a clinically important effect on birth weight. The intervention was introduced under conditions likely to be replicable in similar health systems with access to smoking cessation services with trained advisors. The intervention comprised a package of measures delivered across both maternity services and smoking cessation services. It was designed to be consistent with national guidance,^8^ although the referral threshold for CO monitoring was lower (4 ppm vs 7ppm) in line with evidence published subsequently.^14^ In common with many healthcare interventions, it was not explicitly developed with an underpinning behavioural theory, but analysis of its components indicated that it incorporated a number of behaviour change techniques, including action planning, monitoring and provision of information.^15 19^ Many of these addressed barriers to implementation identified in previous work.^10 20 21^

There were some limitations in our evaluation. We used routinely collected data from a number of different sources. Organisations collected different variables or defined variables differently, and these were combined and unified to provide a single measure of smoking status in pregnancy. Some variables had high levels of missing data.

The study design was by necessity non-randomised and observational, and relied on routinely collected data which varied in definition and completeness in different organisations. Thus, alternative explanations for the findings should be considered. There may have been changes in characteristics of the women over the study period; however, we included important confounding variables in our statistical models, and the before and after periods overlapped in different trusts. The increase in referral rates after implementation of the intervention is likely attributable in part to improved ascertainment, at least in some trusts; indeed, this was an explicit objective of the intervention. However, the primary outcome of smoking at time of delivery was collected across all trusts throughout the study period according to Department of Health requirements. Furthermore, stopping smoking was more likely in women with a recorded referral to smoking cessation services and a recorded quit date, suggesting that the impact of the intervention on quit rates is mediated in part by increased referrals to smoking cessation services.

During the introduction of the intervention, responsibility for smoking cessation services was moved from health services to local government, resulting in delayed implementation which meant that we could not evaluate any additional impact of the ‘risk perception’ element of the intervention. Our findings may not be generalisable to all settings, for example, areas with lower baseline prevalence of smoking during pregnancy. We were unable to determine whether the effect of the intervention was sustained beyond 4 months, or postnatally in individual women; relapse rates are reported to be around 40% postpartum.^22^ We were also unable to quantify unintended positive consequences, such as partners stopping smoking.

The intervention targeted behaviours at a number of levels across the healthcare system, both organisational and individual. It was not possible to identify specific aspects of this complex intervention which led to the observed changes, nor were we able to confirm whether the positive effects we observed were sustained. Our finding that additional training sessions increased referrals suggests that repeated training may be required to prevent attenuation.^23^ Some of the effect of the intervention may be attributable to increased attention, focus and priority on smoking in pregnancy, irrespective of the particular elements unique to this intervention.

### Comparison with other studies

Referrals to smoking cessation services rose progressively after implementation, and this may reflect both the implementation of the explicit referral threshold and pathway, as well as the establishment of formal recording systems within smoking cessation services, which were not universally in place prior to implementation. Increased referrals were accompanied by increased quit rates; furthermore, quit rates were significantly increased where there was a recorded referral to smoking cessation services or a recorded quit date, suggesting that increased referrals resulted directly in higher quit rates.

Two smaller studies of implementation of routine biochemical validation and opt-out referral have been published. The first, in 3700 pregnant women in two hospitals, found that referrals to smoking cessation services increased, but quit rates did not^11^; the authors attributed this to an increase in referrals of women less motivated to quit. The second, in nearly 5000 pregnant women from a single hospital trust, reported a doubling of 4-week quit rates,^12^ similar to the results of our larger multisite study. This may reflect the emphasis on skills training in the latter two studies, with particular focus on communication skills for introducing universal CO monitoring into the routine antenatal consultation. Few other studies have evaluated service models for smoking in pregnancy, despite evidence of low referral rates.^24^ McGowan *et al* evaluated the implementation of routine CO screening and opt-out referral across a number of hospitals, but provided no data on quit rates^21^; furthermore, midwives had difficulties implementing CO screening, an important issue directly addressed within the babyClear training sessions.

The intervention aimed to improve referral rates into NHS smoking cessation services, which have been reported to be effective,^25^ but in recent years, service models have changed in response to reduced budgets. Our reported effect on quit rates is within the range of effect sizes for trials of interventions to promote smoking cessation in pregnancy.^5 6^ Women from deprived areas were less likely to quit, in line with other studies.^26^

There was considerable variation in referrals and quit rates across individual trusts in this study, despite adjustment for key sociodemographic confounders. Variation between trusts may be partly explained by differences in the fidelity of implementation of the intervention. We conducted a parallel qualitative process evaluation aiming to illuminate the process of implementation within participating organisations, which will be reported separately. We used evidence from the qualitative data to develop variables to describe some of the variability in implementation across trusts. Our quantitative results indicate that this variability at the trust level did contribute to differences in the observed referral and quit rates; specifically, trusts that had a formal system for early contact with women, and the provision of additional training sessions, increased referrals. Nonetheless, it is possible that the observed trust level effects could have arisen because of unmeasured variation between trusts, supporting our a priori adoption of a mixed-effects modelling approach.

We found that women who smoked throughout pregnancy delivered infants nearly 260 g lighter at term than non-smokers. This is a bigger effect than reported in some studies,^27 28^ but as in other previous work, we found the effect of smoking on birth weight was largely reversible by quitting during pregnancy.^29^

A wide range of interventions to promote smoking cessation during pregnancy have been estimated to be cost-effective, including individual support from trained advisers typical of that provided within this study.^4 30^ We estimated the cost per delivery of the intervention to be £30; the cost per smoker will vary with local smoking prevalence. Our estimates include the costs of additional workload generated within smoking cessation services from increased referrals, but exclude the costs before introduction of the intervention. Previous economic evaluations^4 30^ have explored the economic implications of the effect of smoking cessation on maternal and infant health outcomes (eg, reduction in pregnancy complications and improvements in birth weight and other birth outcomes). Our economic analysis focussed solely on the costs of implementing the intervention and the consequences in relation to quit rates. Healthcare costs associated with deliveries and changes in health outcomes were not included due to lack of the required data. Given the health service costs and expected health consequences incurred by smoking in pregnancy, the intervention is likely to be cost-effective compared with other smoking cessation interventions^7^ and conventional thresholds for cost-effectiveness adopted in the UK.^31^

### Meaning and implications

This complex intervention, aimed at improving skills, resources and referral pathways across local healthcare systems, provides a feasible and effective method of implementing evidence-based guidelines for smoking cessation in pregnancy. A system-wide focus on routine identification and referral of pregnant smokers is likely to be cost-effective and to have an important impact on pregnancy outcome. Future research should address requirements for sustainability of such an approach, and explore reasons for local variability in implementation success.

The views expressed are those of the authors and not necessarily those of the NHS, the above named funders or the Department of Health.

The funders had no role in study design, data collection, data analysis, data interpretation, writing the report, and decision to submit for publication.

**What this paper adds**

**What is already known on this subject**

- Smoking in pregnancy causes serious harm to the health of the developing fetus, which can be reduced by systematic identification and treatment of pregnant smokers.
- Smoking in pregnancy rates remain high with wide variation by location and socioeconomic position.
- Barriers to effective implementation of evidence-based best practice include lack of resources, skills and motivation of frontline staff.
- Recent research in individual hospitals suggests that implementation of routine carbon monoxide monitoring and opt-out referral of pregnant smokers to smoking cessation services increases referral rates, with unclear impacts on quit rates.

**What this study adds**

- This study provides evidence that implementation of a system-wide intervention across a regional health system focused on skills training, systematic universal carbon monoxide monitoring with opt-out referral, and improved referral pathways and communications between maternity and smoking cessation services can significantly increase referrals and quit rates.
