# Supplementary material for: Evaluation of a complex healthcare intervention to increase smoking cessation in pregnant women: interrupted time series analysis with economic evaluation
Source: Tob Control. 2017 Feb 15;27(1):90–8. doi: 10.1136/tobaccocontrol-2016-053476 (PMC5801649; doi:10.1136/tobaccocontrol-2016-053476)
Supplement: Supplementary file [file tobaccocontrol-2016-053476supp002.pdf]

Appendix 2

Additional data relating to variable definitions, models 1-3 and missing values

Box 2.1: Variable definitions (source of data)

Response variables for smoking behaviour throughout pregnancy	1- Non-smokers (N= 27050) include:<br>- No record of smoking, quitting, smoking cessation service (SCS) contact throughout pregnancy<br>- Record of not smoking consistent throughout pregnancy<br><br>2- Smokers during pregnancy (N= 10676) include:<br>- Recorded smokers at booking (maternity, SCS)<br>- Records of quit date/ having set a quit date<br>- Records of attending/refusing appointment(s) with SCS<br>- Referral/ referral date with SCS<br>- Recorded smoker at booking (maternity, SCS)<br>- Record of information sent or any contact by SCS<br>- Recorded status for quit at 4 weeks' by SCS (any but NA)<br>- Recorded smokers at delivery (maternity)<br><br>From status of smokers at booking, we were able to identify smokers at delivery and those who had quit at delivery:<br>3- Smokers at delivery (N=6882) includes:<br>- Recorded smokers at delivery (maternity)<br>4- Quitters by delivery (N= 3712) includes:<br>- Recorded smokers at booking that are not recorded as smokers at delivery<br>5- Unknown smoking status (N= 82) includes:<br>- Smokers at booking with unknown smoking status at delivery (cannot determine whether they quit or not)
Engagement with smoking cessation services	Setting a quit date (N=881) includes records of having set a quit date(s), one or more quit date(s) recorded, SCS outcome as having set a quit date.
Referrals to smoking cessation services	N=5613 referrals, include all observations with any record with SCS of:<br>- Whether a quit date was set<br>- Whether appointment(s) were attended or booked<br>- Ever being referred to SCS, any referral date<br>- Being sent information or contacted by SCS<br>- Any record of smoking status for 'quit at 4 weeks'
Estimated first appointment date	First appointment reflects the first potential contact within babyClear©; booking dates were not included as a variable in the dataset.

	Estimated conception date = Delivery date – gestational age at delivery<br><br>Estimated first appointment date = Estimated conception date + 11 weeks
Before or after intervention	A response variable is needed that accounts for whether the first booking occurred before or after the start of the intervention. In some cases of first referrals occurring prior to the intervention, further referrals and appointments may have taken place after intervention introduction. This classification considers these pregnancies as "prior" which is to be interpreted as not having received the full intervention protocol.
Strategy used for initial contact and referral with smokers	Developed from information gathered during process evaluation (SJ, personal communication) regarding different approaches to arranging contact between women and smoking cessation services<br><br>Categories:<br><br>None: SCS contacted woman after first appointment with midwife<br><br>Appointment: Referred directly SCS by midwives during the first appointment<br><br>Early contact: A specific enhanced strategy for early contact with smokers before their first appointment with a midwife

Table 2.1: Individual explanatory variables definition

Variable name	Source	Format provided	Unit in analysis	Notes
Foundation Trust	Foundatio n Trust	String	Categorical	Anonymised (A to H)
Maternal age at delivery	Maternity services	Numeric (years)	Categorical (15-20 21-30 31-40 41-55)	
Date of Additional Training		Date String	Month of Training	
Sex of the baby	Maternity services	String	Categorical	Male or Female
Birthweight of the baby	Maternity services	Numeric (grams)	Numeric (grams)	
Gestational age at delivery	Maternity services	Numeric (weeks)	Numeric (weeks)	Completed weeks of gestation
Maternal parity	Maternity services	Numeric (number of births previous registered stillbirths and live births)	Categorical (First child, Second child, Third child or more)	
Maternal weight	Maternity services	Numeric (kg)	Numeric (kg)	*Not used*
Maternal height	Maternity services	Numeric (m)	Numeric (m)	*Not used*
BMI	Maternity services	Numeric (kg/m^2)	Categorical (<20, 20-24.9, 25-29.9, 30)	*Calculated using weight height when provided, if not using value 'recorded'.*
Mother's ethnic origin	Maternity services	Categorical according to national code	Categorical (White non-White)	White British<br>White Irish<br>Any other white background<br>Mixed – White and black Caribbean<br>Mixed – White and black African<br>Mixed - White and Asian<br>Any other mixed background<br>Asian – Indian<br>Asian – Pakistani<br>Asian - Bangladeshi<br>Asian Other

				Black or Black British - Caribbean<br>Black or Black British - African<br>Black or Black British – any other Black Background<br>Chinese<br>Any other ethnic group<br>Not stated
IMD score (Quintiles)	Postcode matching	Numeric, 2007 IMD scores	Categorical (0-16<br>17-64<br>65-80)	Aggregated score and scores for each domain downloaded from: http://data.gov.uk/dataset/index_of_multiple_deprivation_imd_2007

We used an area based measure of deprivation based on lower super output area (LSOA). The LSOA was linked directly to maternal postcode and the overall score for Indices for Multiple Deprivation (IMD) was obtained. IMD scores for each mother were then partitioned into deprivation quintiles by dividing the range of scores into five equal groups. We partitioned the range of IMD scores based on LSOA for the northeast region as a means of generating a measure of deprivation that may be more representative of local area as opposed to national statistics where it has been suggested that the distribution of deprivation may unequal between geographical areas. The IMD quintiles were ranked and reclassified as ordinal variables according to the magnitude of deprivation. Quintiles were reclassified 1-5 from the least deprived to the most deprived scores.

Models

Table 2.2. Estimation of effect sizes and corresponding standard errors, on rate of monthly referral counts for model 1.

Variable	Estimate	Standard Error	Z-value	Pr(>|z|)	IRR	95% CI
Intercept: Baseline	-3.85	0.48	-8.02	<0.0001	0.02	0.01-0.06
babyClear© intervention, compared to before intervention for reference						
First month	0.14	0.08	1.68	0.0934	1.15	0.98-1.35
Second month	0.41	0.08	5.30	<0.0001	1.50	1.29-1.74
Third month	0.76	0.07	11.05	<0.0001	2.14	1.87-2.45
Fourth month and beyond	0.90	0.07	13.50	<0.0001	2.47	2.16-2.81
Time since introduction of intervention, compared to before babyClear© for reference						
Each additional month	0.05	0.03	1.63	0.1031	1.06	0.99-1.13
Strategy for early initial contact with smokers, compared to none						
Appointment	0.58	0.24	2.42	0.0156	1.78	1.12-2.84
Early contact	1.83	0.34	5.36	<0.0001	6.21	3.18-12.11
Month of training, compared to month without training for reference						
On month of training	0.14	0.04	3.48	<0.0001	1.15	1.06-1.25

Generalized linear mixed-effects model of monthly referral counts with a Poisson error distribution and an offset for monthly number of deliveries per trust. The random-effect structure contained a random intercept for trust which allows for heterogeneity between trusts in their baseline number of referrals, and a random slope for time correlated with trust intercept which allows heterogeneity between trusts in the deviation from the overall baseline in referral counts through time. The exponential of the coefficient estimates are used to calculate Incidence Rate Ratio's (IRR). Table 2.3 illustrates the variation between Trusts in the effect on referrals. The Trust Incidence Rate Ratio (IRR) is calculated from random intercept and random slope for each trust, whereby the trust effect IRR is the sum of the IRR from base-line, random intercept and slope.

Table 2.3. Trust effect on referrals.

Incidence Rate Ratio for Each Trust				
Trust	Base-line IRR	Trust IRR	Slope IRR	Trust Effect IRR
A	0.02	2.277	0.935	3.232
B	0.02	2.346	0.951	3.317
C	0.02	1.011	1.020	2.051
D	0.02	0.072	1.193	1.285
E	0.02	1.593	0.960	2.573
F	0.02	0.602	1.052	1.674
G	0.02	5.321	0.871	6.212
H	0.02	0.514	1.050	1.584

Table 2.4: Estimation of effect sizes and corresponding standard errors on probability of quitting by delivery for the full model 2.

Variable	Estimate	Standard Error	z value	Pr(>|z|)	Odds Ratio	95% CI
Intercept: Baseline	-2.05	0.19	-10.66	<0.0001	0.1284	0.09-0.19
After introduction of intervention, compared to before						
After intervention	0.59	0.08	7.35	<0.0001	1.8104	1.55-2.12
Time since start of intervention						
Each additional month	0.02	0.01	2.18	0.0157	1.016	1.00-1.03
Ethnic group, compared to White for reference						
Non white	0.92	0.16	5.75	<0.0001	2.52	1.84-3.44
IMD category, compared to category of IMD scores 17-84 for reference						
[0, 16]: Least deprived	1.01	0.07	13.88	<0.0001	2.75	2.39-3.18
[65, 80]: Most deprived	-0.66	0.11	-5.76	<0.0001	0.52	0.42-0.65
Maternal age category, compared to 21-30yr for reference						
age [15, 20]	-0.28	0.07	-3.94	<0.0001	0.75	0.66-0.87
age [31, 40]	0.36	0.06	6.47	<0.0001	1.43	1.29-1.60
age [41, 55]	0.13	0.21	0.63	0.5251	1.14	0.76-1.71
Engagement with SSS, compared to not referred to SSS for reference						
Referred & quit date	1.43	0.09	16.68	<0.0001	4.18	3.53-4.94
Referred, no quit date	1.20	0.06	21.88	<0.0001	3.33	2.99-3.71

Generalized linear mixed-effects model analysing the probability of quitting by delivery. The model assessed the smoking status of each individual mother at delivery (smoker or quitter) following assessment of smoking status at booking. We used a binomial error distribution. A random intercept for trust which allows for heterogeneity in the baseline probability of quitting by delivery between observations recorded at different trusts.

Table 2.5: Estimation of effect sizes and corresponding standard errors on probability of quitting by delivery for the parsimonious model 2.

Variable	Estimate	Standard Error	z value	Pr(>|z|)	Odds Ratio	95% CI
Intercept: Baseline	-2.05	0.16	-13.22	<0.0001	0.13	0.09-0.17
After introduction of intervention, compared to before						
After intervention	0.59	0.08	7.35	<0.0001	1.80	1.56-2.17
Time since start of intervention						
Each additional month	0.02	0.01	2.18	0.0293	1.01	0.97-1.05
Ethnic group, compared to White for reference						
Non white	0.93	0.16	5.76	<0.0001	2.53	1.84-3.46
IMD category, compared to category of IMD scores 17-84 for reference						
[0, 16]: Least deprived	1.01	0.07	13.91	<0.0001	2.76	2.39-3.18
[65, 80]: Most deprived	-0.65	0.11	-5.71	<0.0001	0.52	0.42-0.65
Maternal age category, compared to 21-30 years for reference						
age [15, 20]	-0.28	0.07	-3.94	<0.0001	0.75	0.66-0.87
age [31, 40]	0.36	0.06	6.43	<0.0001	1.43	1.28-1.60
age [41, 55]	0.14	0.21	0.69	0.4926	1.15	0.77-1.74
Engagement with SSS, compared to not referred to SSS for reference						
Referred & quit date	1.44	0.09	16.48	<0.0001	4.23	3.56-5.02
Referred, no quit date	1.17	0.06	21.01	<0.0001	3.21	2.88-3.58

Table 2.6: Estimation of effect sizes and corresponding standard errors of changes in birthweight (g) for the full model 3.

	Estimated effect size on log(birthweight)				Estimated Change in birthweight (g)	
Variable	Estimate	Standard Error	t-value	Pr(>|t|)	Mean	95% CI
Intercept: Baseline 40wk	8.08	0.00	1810.9	<0.0001	3226.98	3198.88-3255.32
Gestational age at birth, centred at 40 weeks gestational age at birth						
Each additional week past 40	0.05	0.001	71.44	<0.0001	158.12	152.27-164.07
Squared	-0.003	0.00	-49.71	<0.001	-11.94	-12.48 to-11.72
Smoking status at delivery (compared to smoker for reference)						
Non-smoker	0.08	0.00	32.49	<0.001	261.38	243.11-280.28
Quitter	0.06	0.00	18.61	<0.001	212.34	187.45-237.31
Maternal age category, compared to 21-30 year for reference						
age [15,20]	0.003	0.003	1.27	0.3410	13.23	-7.04 to 34.18
age [31,40]	0.001	0.002	0.76	0.4796	4.84	-7.36 to 16.93
age [41,55]	-0.002	0.01	-0.45	0.7511	-9.68	-51.82 to 33.53
Parity (compared to second child for reference)						
First child	-0.03	0.002	-16.45	<0.0001	-105.84	-117.08 to -94.08
Third or more	0.01	0.002	2.74	<0.0067	20.65	5.76-35.81
Ethnicity (compared to White for reference)						
Non White	-0.04	0.003	-13.96	<0.0001	-140.7	-158.34 to -122.4
Deprivation (IMD score compared to 3 central quintiles [17-64] for reference)						
[65-80] Most Deprived	-0.004	0.00	-1.17	0.2917	-15.49	-40.95 to 10.42
[0-16] Least Deprived	0.01	0.00	4.11	<0.0001	28.4	14.71-42.32
BMI (compared to healthy weight for reference)						
Underweight	-0.04	0.01	-7.15	<0.0001	-116.49	-146.51 to -85.61
Overweight	0.02	0.002	11.81	<0.0001	79.06	65.26-93.1
Obese	0.04	0.002	18.58	<0.0001	135.21	117.64-151.05
Sex of baby (compared to Female for reference)						
Male	0.04	0.002	23.2	<0.0001	130.37	118.04-142.58
Effect of Time						
cos(pi*month* 2/12)	-0.002	0.001	-1.34	0.1311	-5.49	-13.44 to 2.6
sin(pi*month* 2/12)	-0.00	0.001	-0.33	0.7414	-1.61	-8.96 to 5.86

A linear mixed-effects model of the log value of weight at birth for each child. A random intercept for trusts allows for heterogeneity in the baseline weight between observations recorded at different trusts.

Table 2.7: Estimation of effect sizes and corresponding standard errors of changes in birthweight (g) for the parsimonious model 3.

	Estimated effect size on log(birthweight)				Estimated Change in birthweight (g)	
Variable	Estimate	Standard Error	t-value	Pr(>|t|)	Mean	95% CI
Intercept: Baseline 40wk	8.08	0.00	1929.04	<0.0001	3233.1	3206.66-3259.75
Gestational age at birth, centred at 40 weeks gestational age at birth						
Each additional week past 40	0.05	0.00	71.58	<0.0001	156.81	151.03-162.66
Squared	-0.00	0.00	-53.43	<0.0001	-12.61	-13.15 to -12.39
Smoking status at delivery (compared to smoker for reference)						
Non-smoker	0.08	0.00	33.35	<0.0001	259.62	241.78-277.73
Quitter	0.06	0.00	18.76	<0.0001	210.15	186.31-235.03
Parity (compared to second child for reference)						
First child	-0.03	0.00	-16.76	<0.0001	-104.43	-115.44 to -92.9
Third or more	0.01	0.00	3.04	<0.0024	22.31	7.70-37.16
Ethnicity (compared to White for reference)						
Non White	-0.04	0.00	-14.24	<0.0001	-141.61	-159.37 to -123.54
Deprivation (IMD score compared to 3 central quintiles [17-64] for reference)						
[65-80] Most Deprived	0.00	0.00	-1.20	0.2316	-15.52	-40.4 to 10.11
[0-16] Least Deprived	0.01	0.00	4.15	<0.0001	27.8	14.43-41.07
BMI (compared to healthy weight for reference)						
Underweight	-0.04	0.01	-7.28	<0.0001	-116.71	-146.54 to -86.38
Overweight	0.02	0.00	12.00	<0.0001	79.53	65.74-93.23
Obese	0.04	0.00	18.74	<0.0001	134.5	119.29-150.27
Sex of baby (compared to Female for reference)						
Male	0.04	0.00	23.24	<0.0001	129.00	117.04-141.5

A quadratic term was used to describe the relationship between gestational age at birth and birthweight. The results show that up to 40 weeks gestation, birthweight increases significantly until a threshold is reached. Beyond this 40-week threshold, birthweight significantly decreases. The linear term in the model, described by each additional week beyond 40 weeks gestation provides an assessment of the increase in birthweight. In contrast, the squared term, which is used additively in the model, describes the downward inflection of the curve and is therefore negative.

Missing values

1. Model of variation in monthly count of referrals

Data were aggregated by month for this analysis, resulting in a total of 132 observation points through time across all trusts 8 trusts. The response variable was concerned with a subset of the population (count of smokers referred), however the model structure included an offset for the monthly sum of observations per trust. In the initial (non-aggregated data), from the total of N=37726 observations, N=36907 were included in the analysis.

From the initial dataset, a total of N=819 records of women were omitted due to either missing records (gestational age) or because information was unavailable to allow initial booking dates to be estimated.

Missing information on gestational age at delivery (N=672, information required to estimate relation to intervention in time, see table 2.7 and 2.8)

Observations during the initial months of data collection in each trust were omitted when information for estimated first booking dates was incomplete (N=41 Table 2.7). Incomplete data during the last four months of estimated first booking date (N=106 observations omitted (Table 2.7).

Table 2.8: Number of records of women omitted from the analyses of monthly referral counts.

Description	Number of Mothers omitted
Gestational age unknown	672
Incomplete information relating to first booking dates	
Beginning of the study period to initiation of study	41
End of the study period	106
Total	819

Table 2.9: Number and percentage of missing records for gestational age at delivery, within each trust and overall.

Trust	Number of Records	Missing Records	Missing Records (% Total)
A	9187	397	4.32%
B	2127	0	0.0%
C	3017	7	0.23%
D	3875	99	2.55%
E	9023	20	0.22%
F	3712	3	0.08%
G	5116	9	0.18%
H	1669	142	8.50%
Total	37726	672	1.78%

Model of probability of quitting by delivery

The number of smokers at booking was N= 10676. This cohort was reduced to N= 9967 observations due to N=709 missing records (Table 2.9).

Table 2.10: Number of observations removed from data for variables used in the analysis of probability of quitting by delivery due to missing records.

Variable	Number of Missing Records
Smoking status at delivery	82
Gestational age at delivery	200
Ethnicity	329
IMD score	131
Maternal age	1
Delivery date	0
Engaged with Stop-smoking Services	0

Model of birthweight

The initial dataset consisted of N=37726 deliveries. This was reduced to N=22826 deliveries due to N=14900 missing records (Table 2.10).

Table 2.11: The number of missing records for birthweight and associated variables.

Variable	Number of missing records
Birthweight >2000g and gestational age < 30wks	8
Birthweight >1000g and gestational age < 20wks	1
Gestational age > 45wks	6
Gestational age < 17wks	13
Gestational age	672
Birthweight	150
Sex	94
IMD score	659
Smoking status	82
Parity	5721
Ethnicity	1376
BMI	12239
Maternal height	12239
Maternal weight	12135
