# Supplementary material for: Evaluation of a complex healthcare intervention to increase smoking cessation in pregnant women: interrupted time series analysis with economic evaluation
Source: Tob Control. 2017 Feb 15;27(1):90–8. doi: 10.1136/tobaccocontrol-2016-053476 (PMC5801649; doi:10.1136/tobaccocontrol-2016-053476)
Supplement: Supplementary file [file tobaccocontrol-2016-053476supp003.pdf]

Supplementary appendix 3

Economic Analysis

Methods

The additional total cost to the NHS of the reconfiguration of services at participating trusts in the North East of England was estimated. In addition, an economic evaluation in the form of a cost-consequence analysis (CCA) and a cost-effectiveness analysis (CEA) was conducted in order to estimate the NHS cost per additional quit.

Cost analysis

For the cost analysis, we estimated costs in three broad areas:

- Cost of training midwives and stop smoking service staff (practitioners) involved in delivery of the babyClear© intervention;
- Cost of implementing the intervention, i.e. investment in equipment, consumables etc.;
- Costs related to changes in workload following implementation of the babyClear© intervention and time spent by practitioners attending training.

Costs were estimated over a five-year time horizon in order to account to account for the costs that would be incurred in subsequent years beyond the initial (more costly) year of implementation (Year one is the first twelve months of implementation at the respective trusts). Costs are presented on a trust-by-trust basis. Whilst eight NHS trusts were involved in the intervention, only six stop smoking services were involved, with Trust B, Trust F and Trust H sharing one stop smoking service. For those costs which are specific to this stop smoking service, costs are shared equally between the three trusts. Costs for an average trust in the North East of England, based upon the mean data of the participating trusts, are also presented. Costs estimated for future years were discounted at a rate of 1.5%, which is appropriate when assessing public health interventions.[1] Yearly costs, total costs for each section and overall costs have been rounded to the nearest pound.

Training of practitioners

As part of the babyClear© intervention, midwifery and stop smoking service staff across all participating NHS trusts were provided with systematic training underpinned by consistent information and effective ways to deliver stop smoking messages to women. The cost of providing training to practitioners was estimated through years one-five of implementation. In year one, total costs of providing training were determined by the contract established with the Tobacco Control Collaborating Centre (TCCC), which was commissioned directly for that purpose.

In subsequent years, the majority of the year one training provision costs would not be incurred as the task of providing training would largely fall under the remit of 'continuous professional development' for staff within the individual trusts. These costs are captured under the heading of 'Changes in workload and time spent attending training'. However, the TCCC would need to be contracted specifically for the purpose of providing risk perception training in subsequent years due to the specialist nature of the training. The cost of contracting the TCCC to provide risk perception training in future years is excluded from this analysis as the quantitative evaluation did not include the risk perception intervention due to delays in implementation across trusts. Therefore, risk perception costs are also excluded from the cost analysis.

Implementing the intervention

The cost of implementing the intervention in year one included the cost of creating information leaflets for the public, the cost of providing midwives with these leaflets and referral pads, the cost of providing trusts with carbon monoxide monitors, amongst other costs. For implementation costs in year one, the costs can be considered fixed as they did not vary greatly by the size of the trust. Therefore, the cost per trust was the average of the total amount. The average trust would incur the same fixed costs in year one.

In subsequent years, it is estimated that 50% of the public facing leaflets required in year one would be needed each year. Consumable costs would also recur on a yearly basis. The cost of consumables in future years will be largely determined by the number of maternities seen in each locality on a yearly basis.

Changes in workload and time spent attending training

The introduction of the babyClear© intervention meant that practitioners (midwives and stop smoking service staff) had to conduct tasks that would otherwise not have been required were babyClear© not introduced. It is necessary to capture these as an economic cost of implementing the babyClear© intervention. The hourly pay rates for NHS workers at the relevant bands were calculated using the NHS pay bands effective from April 2012-April 2013.[2] The mid-point of each band was used to calculate costs. Where there was a mixture of bands, the average salary across bands was used to calculate costs. Salaries were also adjusted to reflect the national insurance and superannuation payments that NHS employers would have made in the relevant year. Relevant hourly staff costs are as follows: Band two/three: £13.18; Band six: £23.46; Band seven: £28.02; and Band six/seven: £25.74.

Hourly pay rates were combined with the time commitment of the various tasks in order to calculate the total cost per trust, and the total cost of the average trust, through years one-five.

Workload

Midwives

For midwifery services, all trusts said that they were already undertaking the three-minute intervention at booking and, so, no further costs were incurred in this regard. Approximately 50% of the trusts also indicated that there was no/not much increase in time spent referring women to stop smoking services following implementation. Therefore, costs associated with increased workload to trusts are primarily related to delivery of the risk perception intervention. Risk perception costs are excluded from this analysis.

Stop Smoking Services

The workload for stop smoking service administrative staff, advisers and leads was impacted by the increase in referral rates following the introduction of the babyClear© intervention. In addition, the increase in referral rates resulted in an increase in the volume of appointment letters and 'failure to attend letters' being sent. The areas of workload which were directly impacted were derived from feedback from the individual stop smoking services. All stop smoking services were contacted to derive estimates of the amount of time that needed to be committed to specific areas as a result of the introduction of the babyClear© intervention, although relatively few data were identified. Therefore, for this cost estimation we looked at the change in the numbers being referred to each stop smoking service from four months before the introduction of babyClear© in each locality and ten months following introduction, based on the data available from the quantitative evaluation. We then estimated the average time commitment required per additional referral using the data derived from the stop smoking services, for each of the different elements of workload which were impacted. This allowed us to estimate costs for all trusts, including those for which there was missing information. Costs for the average trust were estimated in the same way. Year one costs for workload change were discounted for future years at a rate of 1.5%.[1]

Training

The final aspect of change in workload to midwifery and stop smoking services is the time spent by practitioners attending training related to the babyClear© intervention in year one, and in subsequent years. Four different types of training were delivered by the TCCC in year one: one-day risk perception training (excluded from analysis), two-hour babyClear© training, one-day SSS advisor training, and one-day SSS admin training. In subsequent years, risk perception training (excluded from analysis) and babyClear© training would take place on an annual basis. Costs associated with attending (and delivering, in the case of babyClear© training) training have been estimated for years one-five.

Economic Evaluation

A cost-consequence analysis (CCA) was conducted in order to display and relate all relevant costs and consequences of the babyClear© intervention. A CCA considers all the health and non-health benefits of an intervention and reports them in a disaggregated form. In this analysis, a 'balance sheet' of outcomes is presented that decision-makers can weigh up against the costs of the babyClear© intervention. In an additional step, a cost-effectiveness analysis (CEA) was conducted in order to estimate the NHS cost per additional quit. This involved estimating the additional cost per average trust and using this data alongside the additional number of quitters in an average trust following implementation.

Results

Cost analysis

Training of practitioners

The cost of providing training to practitioners across trusts in year one was determined by the contract established with the Tobacco Control Collaborating Centre (TCCC). The total cost of contracting the TCCC to provide training across all trusts in year one was £106,300. In year one, 600 practitioners (midwives and stop smoking service staff) received training. The number of practitioners receiving training in year one varied greatly across trusts. The cost per trust in year one was based on the percentage of the total number trained in the individual trusts (Table 1):

Table 3.1: Cost (£) of providing training per trust in year 1, based on the number of practitioners trained:

Locality	Trust A	Trust B	Trust C	Trust D	Trust E	Trust F	Trust G	Trust H	Average Trust
**Number of practitioners trained**	**144**	28	51	104	55	72	103	43	75
**% of total numbers trained in Year 1**	24%	5%	9%	17%	9%	12%	17%	7%	13%
**Cost (£) per locality**	**25,512**	**5,315**	**9,567**	**18,071**	**9,567**	**12,756**	**18,071**	**7,441**	**13,819**

The cost of providing training to practitioners across trusts in subsequent years is £0.

The total cost per trust of providing training through years one-five is presented in Table 2:

Table 3. 2: Cost (£) of providing training per trust through years 1-5:

	Trust A	Trust B	Trust C	Trust D	Trust E	Trust F	Trust G	Trust H	Average Trust
**Year 1**	25,512	5,315	9,567	18,071	9,567	12,756	18,071	7,441	13,819
**Year 2**	0	0	0	0	0	0	0	0	0
**Year 3**	0	0	0	0	0	0	0	0	0
**Year 4**	0	0	0	0	0	0	0	0	0
**Year 5**	0	0	0	0	0	0	0	0	0
**Total (£)**	**25,512**	**5,315**	**9,567**	**18,071**	**9,567**	**12,756**	**18,071**	**7,441**	**13,819**

Implementing the intervention

Implementing the intervention in year one included the cost of the following items: Public facing leaflets; artwork for leaflets; information leaflets for midwives; referral pads for midwives; babyClear© boxes; carbon monoxide monitors and monitor d-pieces, and consumables. The average cost of implementing the intervention in year one was £7,339 per trust. In subsequent years, it is estimated that 50% of the public facing leaflets required in year one (£187.50 per trust (£1,500/8), discounted for subsequent years) would be needed on an annual basis. The cost of consumables in future years will be largely determined by the number of maternities seen in each locality on a yearly basis. All other costs related to implementation in year one would not recur in future years. Annual costs of consumables for each locality based on the number of maternities seen in each trust prior to the year of implementation are as follows:

- Trust A: £3,000;

- Trust B: £1,000;
- Trust C: £1,500;
- Trust D: £1,200;
- Trust E: £1,500;
- Trust F: £2,000;
- Trust G: £2,500;
- Trust H: £1,000;
- Average trust (approx. 50% of the number of maternities as Trust A): £1,500.

Total costs per trust of implementing the intervention in year one and in subsequent years have been estimated and discounted at a rate of 1.5% per annum (Table 3):

Table 3.3: Implementation costs (£) per trust through year 1-5:

	Trust A	Trust B	Trust C	Trust D	Trust E	Trust F	Trust G	Trust H	Average Trust
**Year 1**	7,339	7,339	7,339	7,339	7,339	7,339	7,339	7,339	7,339
**Year 2**	3,140	1,170	1,663	1,367	1,663	2,155	2,648	1,170	1,663
**Year 3**	3,094	1,153	1,638	1,347	1,638	2,123	2,609	1,153	1,638
**Year 4**	3,048	1,136	1,614	1,327	1,614	2,092	2,570	1,136	1,614
**Year 5**	3,003	1,119	1,590	1,307	1,590	2,061	2,532	1,119	1,590
**Total (£)**	**19,625**	**11,916**	**13,843**	**12,687**	**13,843**	**15,771**	**17,698**	**11,916**	**13,843**

Changes in workload and time spent attending training

Workload

Midwives

Costs associated with increased workload to midwives at trusts are related to delivery of the risk perception intervention. Risk perception costs are excluded from this analysis.

Stop Smoking Services

Yearly costs per trust associated with increased workload to stop smoking services, and the specific areas which were impacted, are presented in Table 4:

Table 3.4: Yearly cost (£) of change in workload to SSS in each trust:

Locality	Trust A	Trust B	Trust C	Trust D	Trust E	Trust F	Trust G	Trust H	Average Trust
**Impact on workload:**									
**Impact on admin hub workload (band 2/3)**	3,954	4,587	20,719	2,214	16,607	4,587	3,796	4,587	8,066
**Increased workload for SSS advisers (band 6)**	25,618	45,606	158,214	16,610	126,402	45,606	28,715	45,606	61,653
**Increased workload for SSS lead (band 7)**	15,803	28,580	98,855	10,423	79,016	28,580	17,821	28,580	38,668

**Increased volume of apt. letters and failure to attend letters**	847	1,512	5,247	552	4,196	1,512	945	1,512	2,049
**Total (year 1) (£)**	**46,222**	**80,285**	**283,034**	**29,799**	**226,221**	**80,285**	**51,277**	**80,285**	**110,435**
**Year 2 (£)**	**45,538**	**79,097**	**278,845**	**29,358**	**222,873**	**79,097**	**50,518**	**79,097**	**108,801**
**Year 3 (£)**	**44,868**	**77,933**	**274,741**	**28,926**	**219,593**	**77,933**	**49,774**	**77,933**	**107,200**
**Year 4 (£)**	**44,202**	**76,777**	**270,666**	**28,497**	**216,336**	**76,777**	**49,036**	**76,777**	**105,609**
**Year 5 (£)**	**43,551**	**75,645**	**266,675**	**28,077**	**213,146**	**75,645**	**48,313**	**75,645**	**104,052**

Training

The number of practitioners trained per trust, and the cost of their time spent attending training, in year one is presented below. The grades of staff attending the respective training sessions is also presented in Table 5:

Table 3.5: Year 1 costs (£) per trust for the time spent by practitioners attending training

Locality	Trust A		Trust B		Trust C		Trust D		Trust E		Trust F		Trust G		Trust H		Average Trust	
	No. trained	Cost (£)	No. trained	Cost (£)	No. trained	Cost (£)	No. trained	Cost (£)	No. trained	Cost (£)	No. trained	Cost (£)	No. trained	Cost (£)	No. trained	Cost (£)	No. trained	Cost (£)
**babyClear© training of midwives (2-hour training) (Band 6/7)**	101	5,199	11	566	31	1,596	73	3,758	45	2,317	53	2,728	73	3,758	26	1,338	52	2,677
**SSS advisor training (1-day event) (Band 6)**	33	5,806	15	2,639	15	2,639	31	5,454	7	1,232	16	2,815	24	4,223	15	2,639	20	3,519
**SSS admin training (1-day event) Band 2/3)**	10	989	2	198	5	494	0	0	3	297	3	297	6	593		198	4	395
**Total (£)**		**11,994**		**3,403**		**4,729**		**9,212**		**3,845**		**5,840**		**8,574**		**4,175**		**6,591**

In subsequent years, it is estimated that approximately 50 midwives (band six/seven) across trusts (figure is an assumption based on the approximate turnover of staff across trusts in the North East) would require two-hour babyClear© training on an annual basis (£2,574 across trusts). As the numbers attending babyClear© training in year one varied greatly across localities, the cost per trust of the time of 50 practitioners spent attending babyClear© training in subsequent years has been estimated based on the year one percentages, i.e. the numbers to receive babyClear© training in each trust in year one as a percentage of the total number to receive babyClear© training in year one (Table 6):

Table 3.6: Costs (£) per trust for subsequent years for time spent attending babyClear© training, based on year 1 percentages:

Locality	Trust A	Trust B	Trust C	Trust D	Trust E	Trust F	Trust G	Trust H	Average Trust
**Year 2**	622	68	190	449	277	324	449	160	319
**Year 3**	613	67	187	443	273	319	443	157	315
**Year 4**	603	66	185	436	269	315	436	155	310
**Year 5**	595	65	182	430	265	310	430	153	305
**Cost (£) before discounting**	**631**	**69**	**193**	**456**	**281**	**329**	**456**	**162**	**324**

The TCCC would not need to be contracted to provide core babyClear© training in future years. Core babyClear© training could be delivered in subsequent years by stop smoking service staff (band six), and it is estimated that nine practitioners would need to commit two hours on two days per year each to deliver this training, based on the assumed numbers requiring training and the assumed numbers of staff that would be required to deliver this training. The average annual cost of this time has been calculated per trust (£106) for future years, and discounted at a rate of 1.5% (Table 7):

Table 3.7: Average costs (£) per trust for subsequent years for time spent delivering babyClear© training:

Subsequent years	Year 2	Year 3	Year 4	Year 5
Cost (£) of 9 practitioners (band 6) per year delivering babyClear© training	104	103	101	100

Total Costs

The total cost per trust of introducing the babyClear© intervention through years one-five is presented in Table 8.

Table 3.8: Total cost (£) per trust:

Locality	Trust A	Trust B	Trust C	Trust D	Trust E	Trust F	Trust G	Trust H	Average trust
**Year 1**	91,068	96,343	304,670	64,422	246,972	106,221	85,261	99,241	138,185
**Year 2**	49,405	80,439	280,803	31,279	224,917	81,681	53,719	80,531	110,887
**Year 3**	48,678	79,256	276,670	30,819	221,607	80,479	52,928	79,346	109,255
**Year 4**	47,955	78,080	272,565	30,361	218,319	79,285	52,143	78,169	107,634
**Year 5**	47,248	76,929	268,547	29,914	215,100	78,116	51,374	77,016	106,047
**Total (£)**	**284,354**	**411,046**	**1,403,254**	**186,794**	**1,126,916**	**425,780**	**295,426**	**414,303**	**572,009**

The total cost of implementing the babyClear© intervention for the average trust in the North East of England through years one-five was £572,009.

Economic Evaluation

Cost-Consequence Analysis

For the cost-consequence analysis, the cost component was the cost of implementing the babyClear© intervention in an average trust in the North East of England. The effects are taken directly from the main effectiveness analysis.

Table 3.9: Balance sheet of costs and outcomes of the intervention (based on cost per average trust in the North East of England following the implementation of babyClear©, and data compiled during the quantitative evaluation):

Favours current practice	Favours babyClear©
**Cost data:**<br>***Implementing babyClear© results in following additional costs:***<br><br>*Training:*<br>Year 1: £13,819, £0 in subsequent years<br><br>*Implementation:*<br>Year 1: £7,339, £1,500-£1,700 per year in subsequent years; *£13,843 in total over 5 years*<br><br>*Change in workload and time spent attending/delivering training:*<br><br>Increased workload: Year 1: £110,435 and similar cost in subsequent years; £536,097 in total over 5 years<br><br>Cost of time spent attending/delivering training: Year 1: £6,591 and £400-£425 in subsequent years; in total £8,248 over 5 years	**Outcome data:**<br>2.47 (2.16-2.81) fold increase in referral counts beyond the fourth month following the introduction of babyClear©, compared to the period before introduction.<br><br>Additional training sessions result in an increase in referrals in the month of training (aOR 1.15 [95% CI 1.06-1.25])<br><br>1.81 (1.54-2.12) fold increase in the probability of quitting by delivery following the introduction of babyClear©.<br><br>Odds of quitting were 3.23 (2.98-3.70) fold higher for deliveries with a recorded referral to smoking cessation services.<br><br>**Cost data:**<br>The cost of treating the consequences of smoking during pregnancy to mother and child can be substantial. An intervention such as babyClear©, which has resulted in an increased probability of quitting by delivery, has the potential to generate costs savings from an NHS perspective.

aOR = adjusted odd ratio

The introduction of babyClear© resulted in a total cost of £572,009 for the average trust in the North East of England, through years one-five of implementation. These additional costs should be considered alongside the favourable outcome data. A judgement is required as to whether these improved outcomes are worth the additional cost of rolling out and sustaining such an intervention.

Cost-Effectiveness Analysis

A cost-effectiveness analysis was also conducted in order to compare the costs and health effects of the intervention and to assess the extent to which it can be regarded as providing value for money.

For the estimation of the incremental cost per additional quitter, the cost of adopting babyClear© in the average trust in the North East of England over five years was £572,009. Over this same time period there were an estimated 3728 deliveries per year in an average trust in the year prior to implementation (2011-12). Thus, over the five year time horizon the total number of deliveries was 18,640. Of these, 28% were smokers.

Without babyClear© the expected quit rate was 0.0398 of the whole number of deliveries (table 9). The quit rate following the introduction of babyClear© was 0.072038. The difference in quit rate was 0.032238.

The additional cost per delivery in an average trust was £30.69 (572009/18640) and the incremental cost per additional quitter is £952 (30.69/0.032238). The number needed to treat to get one additional quitter is 31 (1/0.032238) pregnant women, or 9 pregnant smokers.

Table 3.10: Baseline proportion of women quitting by delivery

Population	N per smoking status				Total N	Proportion (%) of quitters by delivery, amongst whole population	Proportion (%) of quitters by delivery, amongst smokers at booking
	non-smoker	quitter	smoker	NA			
Trust A	1385	103	375	14	1877	5.49	21.55
Trust B	446	30	71	3	550	5.45	29.70
Trust C	693	64	172	0	929	6.89	27.12
Trust D	1114	32	285	1	1432	2.23	10.09
Trust E	2358	94	346	0	2798	3.36	21.36
Trust F	709	33	199	0	941	3.51	14.22
Trust G	1457	65	401	0	1923	3.38	13.95
Trust H	298	11	97	0	406	2.71	10.19
Total	8460	432	1946	18	10856	3.98	18.17

References

1. Methods for the development of NICE public health guidance (third edition), 2012. https://www.nice.org.uk/article/pmg4/chapter/appendix-i-quality-appraisal-checklist-economic-evaluations.
2. Royal College of Nursing. 2015. *Pay rates 2012/13*. [ONLINE] Available at: https://www.rcn.org.uk/support/pay_and_conditions/pay_rates_201213. [Accessed 01 December 15].
